# Sex differences in olfactory cortex neuronal loss in aging

**DOI:** 10.3389/fnhum.2023.1130200

**Published:** 2023-05-31

**Authors:** Majed M. Alotaibi, Matteo De Marco, Annalena Venneri

**Affiliations:** ^1^Sheffield Institute for Translational Neuroscience, The University of Sheffield, Sheffield, United Kingdom; ^2^Department of Medical Genomics Research, King Abdullah International Medical Research Center, King Saud bin Abdulaziz University for Health Sciences, Riyadh, Saudi Arabia; ^3^Department of Life Sciences, Brunel University London, Uxbridge, United Kingdom; ^4^Department of Medicine and Surgery, University of Parma, Parma, Italy

**Keywords:** aging, sex, atrophy, olfactory cortex, neuroimaging, neuronal loss

## Abstract

**Introduction:**

Aging plays a major role in neurodegenerative disorders such as Alzheimer’s disease, and impacts neuronal loss. Olfactory dysfunction can be an early alteration heralding the presence of a neurodegenerative disorder in aging. Studying alterations in olfaction-related brain regions might help detection of neurodegenerative diseases at an earlier stage as well as protect individuals from any danger caused by loss of sense of smell.

**Objective:**

To assess the effect of age and sex on olfactory cortex volume in cognitively healthy participants.

**Method:**

Neurologically healthy participants were divided in three groups based on their age: young (20–35 years; *n* = 53), middle-aged (36–65 years; *n* = 66) and older (66–85 years; *n* = 95). T1-weighted MRI scans acquired at 1.5 T were processed using SPM12. Smoothed images were used to extract the volume of olfactory cortex regions.

**Results:**

ANCOVA analyses showed significant differences in volume between age groups in the olfactory cortex (*p* ≤ 0.0001). In women, neuronal loss started earlier than in men (in the 4th decade of life), while in men more substantial neuronal loss in olfactory cortex regions was detected only later in life.

**Conclusion:**

Data indicate that age-related reduction in the volume of the olfactory cortex starts earlier in women than in men. The findings suggest that volume changes in olfaction-related brain regions in the aging population deserve further attention as potential proxies of increased risk of neurodegenerative diseases.

## 1. Introduction

The process of aging has an effect on the regional integrity of the human brain via alterations in levels of hormones, neurotransmitters and neurotrophic factors (Peters, 2006). These aging effects may alter the size as well as the composition of the neural tissue, and are the outcome of a combination of contributing factors such as sex, education and structural biological factors. The impact of these aspects is of clinical interest because they are linked to the mechanisms at the basis of the development of diseases or conditions that might affect individuals’ ability to lead a normal life. However, there is interindividual variation in how different brain regions may be susceptible to disease and how these undergo atrophy (Grajauskas et al., 2019). Importantly, not every brain region is altered to the same extent by the presence of pathology (Trollor and Valenzuela, 2001). Aging plays a major role in fostering neurological disorders such as Alzheimer’s disease (AD), cardiac/cerebrovascular disease, or other pathologies that cause cognitive decline (Cermakova et al., 2015; Liesinger et al., 2018). Neurodegeneration can also lead to olfactory dysfunction that can manifest very early and, in some cases, herald the presence of a neurodegenerative disorder, such as Alzheimer’s disease (Gray et al., 2001; Sohrabi et al., 2012; Devanand et al., 2015). There is, in fact, evidence of early pathological changes, such as β-amyloid and neurofibrillary tangles, in regions responsible for olfactory functioning (Christen-Zaech et al., 2003; Attems and Jellinger, 2006). There also is evidence, however, of olfactory decline during normal aging, in the absence of neurodegeneration (Wang et al., 2016). In addition, olfactory dysfunction has been found to be associated with volume reduction in the olfactory cortex (OCV) and the hippocampus (Bitter et al., 2010) that, in addition to memory function, is also involved in aspects of olfactory processing.

Sex differences have been detected in how olfaction changes over time. Women have higher activation in olfactory regions than men during normal processing (Yousem et al., 1999). Furthermore, women are at an increased risk of AD and have faster brain atrophy in the hippocampus than men (Mazure and Swendsen, 2016; Nebel et al., 2018). Overall, a solid body of studies indicates that disease progression is more rapid among women, and that women, on average, also show longer disease durations (Malpetti et al., 2017; Liesinger et al., 2018). In addition, women also show greater neurovolumetric reduction than men during their fourth and fifth decade of life (Guo et al., 2016) that contributes to the definition of sex-specific trajectories in neurological aging.

The number of studies of the olfactory cortex in aging women is small. Published evidence indicates that women retain integrity of olfactory regions but show volumetric reduction in subsidiary areas involved in secondary olfactory processing, such as the hippocampus, parahippocampus, entorhinal cortex, amygdala and orbitofrontal cortex (Tamnes et al., 2013; Jiang et al., 2014; Sele et al., 2020).

Understanding and measuring volumetric loss in the olfactory cortex and in structures that support olfactory processing is important since these regions are also involved in Alzheimer’s disease, the most common neurodegenerative disease, for which an early olfactory dysfunction might be the reflection of an increased aging-related risk for decline (Attems and Jellinger, 2006; Kocahan and Doğan, 2017). Olfactory loss is also found in other forms of neurodegeneration such as Parkinson’s disease (Rahayel et al., 2012). Studying alterations in the olfactory cortex might help detection of neurodegenerative diseases at an earlier stage than currently done.

The present study used Magnetic Resonance Imaging (MRI) to acquire brain scans to determine the extent of volume loss in olfactory cortex in the course of normal aging. Volumetric measurements were acquired in three age groups, young adults, middle-age adults and older adults, all neurologically healthy, to determine the level of regional neural volume loss related to physiological aging. In light of the sex-specific differences outlined above, we expected to find that women would present with an earlier olfactory cortex volume reduction than men.

## 2. Materials and methods

### 2.1. Participants

Neurologically healthy participants were selected from the cohort of volunteers who had participated as healthy controls in several MRI based research projects coordinated by the Department of Neuroscience at the University of Sheffield (UK). These cognitively healthy adult participants were between 20 and 85 years of age. In total, 333 datasets were retrieved and considered for inclusion in this study. The resulting age-based groups were classified as “young”, i.e., aged 20–35 (*n* = 53), “middle-age”, i.e., aged 36–65 (*n* = 66) and “older”, i.e., aged 66–85 (*n* = 93). Clinical profiling via comprehensive neuropsychological testing was available for those participants who were part of the middle-age and older groups, and were 40 years old and older, to confirm their cognitive clinical status as unimpaired (i.e., absence of a cognitive profile was the primary reason for excluding about one third of the datasets originally taken into consideration). This comprehensive testing has been described in detail in previous publications (De Marco et al., 2019, 2017) and the mean scores of the samples included in this study are shown in Table 1. All eligible participants who had completed a 1.5 T MRI protocol inclusive of a three-dimensional T1-weighted brain image and had a complete neuropsychological assessment were included. All procedures were carried out in compliance with the Declaration of Helsinki. Written consent was obtained from all participants. Ethical approval and Health Research Authority approval for retrospective data analyses were obtained from the West of Scotland Regional Ethics Committee 5, Ref No.: 19/WS/0177.

**TABLE 1:** Neuropsychological profile of middle-aged and older adults.

Test	Middle-aged	Older
Mini Mental State Examination	29.21 (1.13)	28.44 (1.58)
Raven’s Colored Progressive Matrices	32.97 (2.58)	28.77 (4.42)
Letter Fluency Test	39.88 (11.46)	33.05 (11.43)
Category Fluency Test	45.59 (7.45)	39.11 (9.65)
Digit Cancellation Test	55.33 (4.05)	51.63 (6.78)
Similarities (WAIS)	21.52 (3.78)	20.47 (4.62)
Token Test	34.92 (1.39)	34.01 (2.23)
Rey-Osterrieth Complex Figure–copy	33.65 (3.00)	32.38 (4.22)
Rey-Osterrieth Complex Figure–recall	18.05 (6.11)	14.20 (5.52)
Stroop Test–time interference	17.86 (6.94)	25.14 (10.58)
Stroop Test–error interference	0.29 (0.58)	1.20 (2.91)
Digit Span Test–forward	6.03 (0.72)	5.92 (1.04)
Digit Span Test–backward	4.35 (0.95)	4.15 (1.00)
Prose Memory Test–immediate recall	11.35 (3.71)	9.73 (3.54)
Prose Memory Test–delayed recall	15.12 (4.65)	13.21 (4.87)
Paired Associated Learning Test	14.52 (3.97)	11.49 (3.60)
Confrontation Naming Test	19.35 (0.98)	18.76 (1.72)

Means and standard deviations are indicated.

### 2.2. MRI processing

Pre-processing of MRI images was carried out using the most updated version of the Statistical Parametric Mapping software package (SPM), i.e., version 12 (Wellcome Centre for Human Neuroimaging, London, UK), running in a Matlab R2016b environment, version 9.1 (Mathworks Inc., Natick, MA, USA). The images were segmented using the “*new segment”* procedure in SPM12 and the overall 3D image of the brain was subdivided into three tissue-classes by separating the intracranial voxels of interest into gray matter, white matter, and cerebrospinal fluid (CSF). Segmented sub-maps of gray matter were then normalized, modulated, and smoothed with an 8-mm Gaussian kernel.

### 2.3. Extraction of olfactory region volume

In order to extract and calculate individual total intracranial volumes, tissue-class volumes were individually extracted in SPM12 using the individuals’ images containing the segmentation parameters (Malone et al., 2015). Gray-matter volume, white-matter volume and CSF volume were computed in liters and converted to milliliters to be compatible with the unit of measurement of olfactory brain region volumes during data analysis. Summing the volume of the three sub-maps was carried out to calculate each individual’s total intracranial volume (TIV).

**FIGURE 1:**
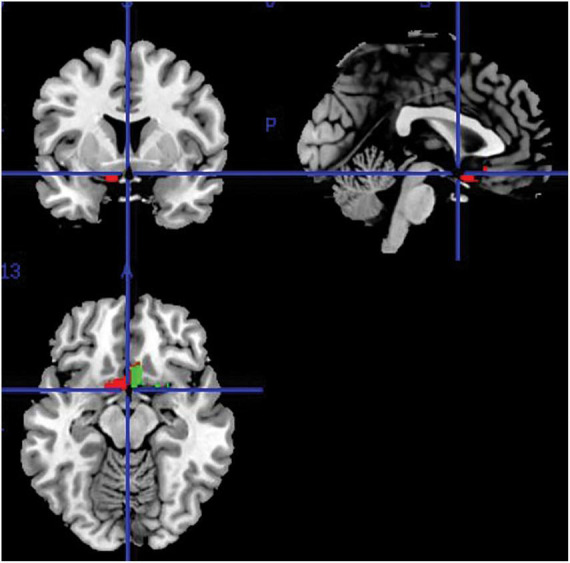
Areas representing the olfactory cortex selected for volume extraction.

Regional volumes (in “ml”) were extracted from all segmented, modulated and smoothed gray-matter images using the “get_totals”^1^ Matlab function to obtain a measurement of the olfactory brain regions of interest (i.e., thus limited to gray-matter tissue only). These were defined via the SPM12 toolbox Wake Forest University (WFU) PickAtlas (Maldjian et al., 2003) that was used to specify olfaction-related regions from a human-brain atlas. This was the Automated Anatomical Labelling (AAL) atlas that was used as the reference to create region-of-interest (ROI) masks for each individual region in the MNI space (Tzourio-Mazoyer et al., 2002). Automatic extraction was done separately for each brain hemisphere. The outcome of this method was separate volumetric measures for the left and right ROI. Summing the ROI volumes from both hemispheres served to obtain the total ROI volume. Figure 1 shows the region selected for extraction. Although this region represents a single entry of the AAL atlas (i.e., the “olfactory cortex” ROI atlas label), subsequent research has shown that it consists of a complex functional territory responsible for olfactory processing that includes the olfactory tract, the amygdala, the piriform cortex, the anterior perforated substance, the subcallosal area and the anterior cingulate cortex, as per definition of the olfactory cortex provided by a recent study (Fjaeldstad et al., 2017).

### 2.4. Data analysis

Statistical data analyses were carried out using the SPSS suite, version 26 (IBM SPSS Statistics for Windows, IBM Corp., Armonk, NY, USA) and the latest version of GraphPad Prism 9 (GraphPad Software San Diego, CA, USA). Descriptive statistics were run to characterize the study population, as shown in Table 2. Initially, the data were analyzed with a 2-by-3, sex-by-age group, factorial *ANOVA*, to test for the interaction effect between the two predictors. The analyses were then stratified by sex: one-way *ANCOVA*s were run to compare the volume of the selected brain region of interest among the three age groups, regressing out the influence of education and TIV. Shapiro–Wilk and Kolmogorov–Smirnov test significance levels, together with the inspection of the histograms illustrating the frequency distribution of outcome variables, were considered as part of the diagnostic process to verify if the ROI volumes were normally distributed. Pearson’s correlation coefficient was used to investigate the association between age, education and TIV, and each of the volumetric measures. The statistical threshold to define the significance level for the ROI volume comparisons was set at *p* < 0.001.

**TABLE 2:** Demographic characteristics of the participants stratified by sex.

	Females			Males		
		**Mean (SD)**			**Mean (SD)**	
	*N*	Age	Education	*N*	Age	Education
Young	33	25.7 (3.8)	16.9 (1.9)	20	26.9 (4.8)	16.5 (3.4)
Middle-age	39	54.0 (8.1)	13.7 (5.3)	27	53.4 (9.2)	13.8 (4.4)
Older	57	72.4 (4.3)	10.3 (4)	38	73.4 (4.9)	12.7 (4.8)

Age and education are indicated in years.

## 3. Results

Significant differences in education were found among the three groups (both sexes: *p* = 0.0001, females: *p* = 0.0001, and males: *p* = 0.009). Significant differences were found for all group comparisons in females, namely young vs. middle-age (*p* = 0.003), young vs. older (*p* < 0.001) and middle-age vs. older (*p* < 0.001). In males, significant differences were found only in the young vs. older (*p* = 0.006) comparison. As expected, significant group differences were found for age (both sexes: *p* = 0.0001, females: *p* = 0.0001, and males: *p* = 0.0001).

### 3.1. Olfactory cortex volume

**FIGURE 2:**
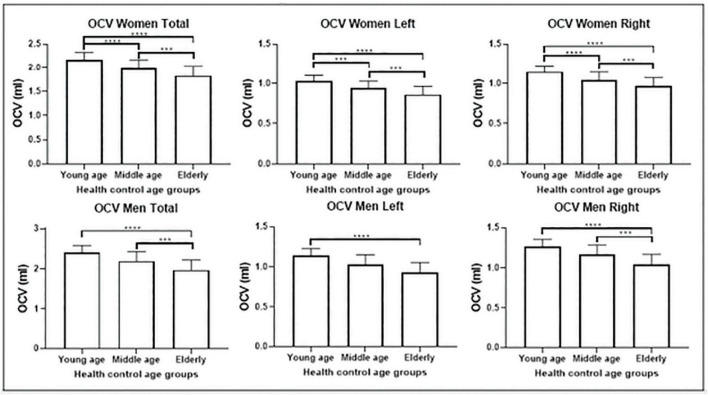
Olfactory cortex volume differences among the three groups: comparisons among the three groups are shown for the female population **(above)** and male population **(below)** separately. Females show atrophy earlier in life than males. ***p < 0.001; ****p < 0.0001.

Olfactory cortex volumes were normally distributed. The two-way *ANOVA* revealed no statistically significant interaction between sex and age group, neither on the volume of the bilateral ROI (*p* = 0.458), nor in the left (*p* = 0.538) or right hemisphere (*p* = 0.370). One-way *ANCOVA* analyses showed significant differences in volume among age groups in OCV (*p* < 0.0001), with a trajectory indicating a negative association with age (Figure 2). *Post-hoc* tests (appropriately corrected for multiple comparisons) showed significant differences in regional volumes across all age groups in the total, left and right OCV in the female population. In men, significant differences were found only between young and older adults for all extracted olfactory cortex volumes and between middle-age and older adults only in the total and right volumes (Figure 2 and Tables 3, 4 for female and male participants, respectively). Left volumes did not differ between middle-age and older males, although a trend approaching significance emerged from this comparison.

**TABLE 3:** Significant differences in volume of the olfactory regions observed among female age groups.

Female		Young vs. middle mean diff. (*p*-value)	Young vs. older mean diff. (*p*-value)	Middle vs. older mean diff. (*p*-value)
Olfactory cortex	Total *F* = 37.3 (*p* < 0.0001)	0.19 (<0.0001)*	0.34 (<0.0001)*	0.15 (0.0003)*
	Left *F* = 35.3 (*p* < 0.0001)	0.09 (0.0001)*	0.16 (<0.0001)*	0.07 (0.0003)*
	Right *F* = 37.8 (*p* < 0.0001)	0.10 (<0.0001)*	0.18 (<0.0001)*	0.08 (0.0003)*

*Corrected threshold of significance of *p* = 0.001.

**TABLE 4:** Significant differences in volume of the olfactory regions observed among male age groups.

Male		Young vs. middle-age mean diff. (*p*-value)	Young vs. older mean diff. (*p*-value)	Middle-age vs. older mean diff. (*p*-value)
Olfactory cortex	Total *F* = 24.1 (*p* < 0.0001)	0.21 (0.01)	0.43 (<0.0001)*	0.21 (0.0009)*
	Left *F* = 22.1 (*p* < 0.0001)	0.11 (0.004)	0.21 (<0.0001)*	0.09 (0.004)
	Right *F* = 25.03 (*p* < 0.0001)	0.10 (0.01)	0.22 (<0.0001)*	0.12 (0.0003)*

*Corrected threshold of significance of *p* = 0.001.

Pearson’s correlation analyses showed a significant link between OCV and age, education and TIV for both sexes (*r* = −0.586, *r* = 0.359 and *r* = 0.619, respectively), in females (*r* = −0.629, *r* = 0.311 and *r* = 0.470, respectively) and in males (*r* = −0.641, *r* = 0.401 and *r* = 0.654 respectively, Table 5). Additional analyses showed that this significant association persisted in all groups when controlling for education (females: *p* = 0.0001, males *p* = 0.0001) or for TIV (females: *p* = 0.0001, males: *p* = 0.0001) separately, and the effect retained its significance in the pairwise tests (Table 5). The statistical comparison of demographic and neurostructural variables between males and females (regardless of age) is included in Supplementary Table 1.

**TABLE 5:** Associations of age, education, and TIV with olfactory cortex.

		Age		Education		TIV	
		***R* ^2^**	***p*-value**	***R* ^2^**	***p*-value**	***R* ^2^**	***p*-value**
OCV	Both sexes	0.3435	<0.0001	0.1288	<0.0001	0.384	<0.0001
	Women	0.3957	<0.0001	0.09653	0.0003	0.2211	<0.0001
	Men	0.4104	<0.0001	0.161	0.0001	0.428	<0.0001

## 4. Discussion

This cohort study investigated volumes of the olfactory cortex and of subsidiary areas involved in secondary olfactory processing, and their associations with sex, age, education and TIV in neurologically healthy adults, via the use of MRI imaging techniques. The findings indicate that women show greater OCV reduction than men and at an earlier age than men. Our study showed that loss of volume in olfactory cortex in women occurs approximately around the fourth decade of life, while in men it is only detectable as late as the seventh decade. This finding is aligned with what has been found previously in longitudinal studies that indicated that women showed significantly greater brain atrophy than men in the fourth and fifth decades of their life (Guo et al., 2016). There is an association between age, sex, and overall brain atrophy rate that appears to be faster in women (Hua et al., 2010). It has been suggested that these sex-based brain alteration differences may be the outcome of more basic differences between men and women that may be due to a variety of causes, such as, for example, differences in hormones, brain development, inflammation, or psychosocial stress response (Toga and Thompson, 2003; Koss and Frick, 2017; Mancini et al., 2017). Modifiable lifestyle factors may also play an important role, as we have previously found that in women who are ex-smokers, olfactory memory is associated with severity of cognitive decline (Alotaibi et al., 2022).

Atrophy in the olfactory cortex in the elderly may predict future illness. A recent study has shown that olfactory dysfunction is detected in a high percentage of elderly participants (Loudghi et al., 2019). Additionally, olfactory dysfunction has been observed in a good proportion of patients with Alzheimer’s disease and some researchers have even suggested that it might represent an early symptom of this disease (Doty, 2009; Wilson et al., 2009; Murphy, 2019). Moreover, some studies indicated that olfactory-related brain regions such as the olfactory bulb, the olfactory tract, the olfactory epithelium, the olfactory cortex and the entorhinal cortex are all significantly atrophic in AD (Devanand et al., 2007; Buschhüter et al., 2008; Thomann et al., 2009; Arnold et al., 2010; Al-Otaibi et al., 2020). The territory responsible for olfactory functioning is particularly susceptible to proteinopathies, including amyloidosis and tauopathy (Ubeda-Bañon et al., 2020), and it is anatomically contiguous to the mediotemporal regions responsible for episodic memory processing that are prominently affected by neurodegenerative processes in AD. This makes olfactory functioning a mechanistically suitable candidate of interest for the detection of those abnormalities triggered by AD. In addition, olfactory testing is feasible in clinical settings (Su et al., 2021) and is typically well-received by testees. Furthermore, investigating abnormal functioning in a clinical population may allow researchers to consolidate the neuroscientific frameworks of olfactory (and, more generally, sensory) perception at its various stages, for instance by distinguishing the mechanisms of perception from those of smell recognition and retrieval of smell-related semantic knowledge. The use of functional MRI could be particularly informative to this end, as it would allow the separation of these computational stages with *ad hoc* experimental manipulations. Similarly, expanding the study of anatomical properties of olfactory regions via other methodologies (e.g., cortical thickness and structural covariance) could provide additional evidence that is, to some degree, complementary to that informed by regional volumes. Resorting to multiple approaches could help shed light on trajectories of neuroanatomical aging. For instance, some researchers have found that gray matter increases until it reaches the plateau in the third decade of life, while other studies have reported that a decrease in volume initiates after the second decade of life (Raz et al., 2004; Shen et al., 2013).

Education has been previously found to be associated with gray matter volume (Boller et al., 2017; Kang et al., 2021), although longitudinal evidence appears to indicate that it has no specific effect on atrophy rates (Nyberg et al., 2021). Moreover, the findings described in these voxel-based studies do not reveal any association between educational attainment and the anatomical integrity of the olfactory cortex. Our results point at a significant association between years of education and the volume of the olfactory cortex in both females and males, but these models were uncorrected for age or TIV, and, for this reason, indicate a general, rather than a specific association. There is evidence that education is associated with better retained cognitive performance in aging, particularly memory performance when this was measured several decades after school discontinuation (Schneeweis et al., 2014), a finding that is in line with the hypothesis that education contributes to better cognitive reserve (Stern, 2009). However, higher levels of education may also contribute to foster brain reserve by limiting brain atrophy and, in turn, resulting in better cognitive performance. Additionally, there is also evidence that functional brain network decline in older adults without college education is greater than older adults with college education (Chan et al., 2021).

The anatomy of olfactory functioning spans across multiple brain regions. The olfactory cortex is highly connected to the orbitofrontal cortex (Du et al., 2020). The olfactory cortex, moreover, includes part of the entorhinal cortex as well as part of the amygdala (Price, 1985; Carmichael et al., 1994; Seubert et al., 2013). These, in turn, are in direct connection with the hippocampus and the parahippocampal cortex (Naya, 2016; McDonald and Mott, 2017). Odorant memory retrieval and emotional activation are supported by the hippocampus and the amygdala (Rolls, 2001; Kubota et al., 2020), while the processes of recall and recognition are sustained by the hippocampal/parahippocampal complex and the orbitofrontal cortex (Rolls, 2001; Iizuka et al., 2021). This shows how important it is to monitor OCV changes in aging, because this region is involved in processing olfaction and emotion, it is connected to memory-related regions, and could be the object of a working hypothesis related to potential mechanisms of detection of neurodegenerative diseases such as AD. Fine-grained inspection of atrophy patterns that extend from the mediotemporal lobe to the medial prefrontal regions that are functionally connected with the primary olfactory cortex (Zhou et al., 2019) in particular, could be a viable clinical procedure that leads to suspecting abnormal olfactory functioning. This could be added to the clinical diagnostic algorithms currently in place to detect the presence of neurological abnormalities in middle-aged and older adults.

Some limitations should be acknowledged. In the literature, different terms are used to indicate the olfactory cortex and, similarly, the anatomical definition of the olfactory cortex is not uniform. Some state that the olfactory cortex includes the piriform cortex (also called pyriform or prepyriform cortex) that is the largest sub-region deputed to olfactory processing (Wilson, 2009). Others define the primary olfactory cortex as including the anterior olfactory nucleus, the pyriform cortex, the periamygdaloid and amygdala complex region, and the rostral entorhinal cortex (Doty, 2017). Others consider the primary olfactory cortex as including the piriform cortex, the anterior olfactory nucleus, the anterior perforated substance, the olfactory tubercle, the anterior portion of periamygdaloid cortex and the amygdala (Wang et al., 2010). In this study, we used the definition of olfactory cortex originally proposed by Dejerine (1895) and used recently in another study (Fjaeldstad et al., 2017), and this includes the olfactory tract, the amygdala, the piriform cortex, the anterior perforated substance, the subcallosal area and the anterior cingulate cortex. This more comprehensive definition might have facilitated the emergence of sex-related differences in these structures, and might have helped highlight a more precise pattern of associations between the predictors and regional volumes. In this respect, this line of research would greatly benefit from the definition of methodological gold standards that can inform researchers on the most adequate choices, e.g., the use of specific ROIs or the inclusion of certain correction factors in the analyses. In addition, it is still possible that the pattern of findings may have been affected by intervenient variables that were not part of the study design. Epidemiological evidence indicates that olfactory dysfunction is influenced by modifiable lifestyle aspects such as exposure to air pollution and toxins, and smoking habits (Yang and Pinto, 2016). As far as chronic smoking is concerned, however, this is not linked to atrophy in the regions responsible for olfactory processing (Sutherland et al., 2016). It is still possible, however, that other mechanisms might be at play in accounting for at least a portion of the findings described in this study. A further limitation in our study, finally, is the absence of data from a validated test of olfactory functioning (e.g., based on olfactory discrimination or recognition).

In conclusion, the data of the current study suggest that age-related reduction in the volume of the olfactory cortex is detectable earlier in women than in men. The findings suggest that monitoring volume changes in olfaction-related brain regions in the aging population may be valuable to detect risk of neurodegenerative diseases, especially in individuals at greater risk. Additionally, more studies are needed to establish a causal link between changes in olfactory functioning and pathological alterations in olfactory cortex during the preclinical phase of AD. This would promote the design of hypothesis-based clinical studies with the purpose of defining clinical guidelines that can help people recognize preclinical abnormalities that could be further investigated by secondary-care specialists. These changes would emerge alongside those that have already been established in other areas of psychological functioning such as semantic memory or perceptual speed (Amieva et al., 2008; Payton et al., 2020) and would help us define the timeline of the olfactory cascade (i.e., when changes in behavioral functioning occur in relation, for instance, to regional atrophy), contributing to outlining a more general and multidimensional profile of risk.

## Data availability statement

The raw data supporting the conclusions of this article will be made available by the authors, without undue reservation.

## Ethics statement

The studies involving human participants were reviewed and approved by the West of Scotland Regional Ethics Committee 5, Ref. No: 19/WS/0177. The participants provided their written informed consent to participate in this study.

## Author contributions

MMA carried out data analysis and wrote the first draft of the manuscript. MDM contributed to the study design, data analysis, and critical review of the manuscript. AV conceived and designed the study, contributed to the data acquisition, critical review, and finalization of the manuscript. All authors approved the submitted version of this manuscript.

## Footnotes

1
http://www0.cs.ucl.ac.uk/staff/g.ridgway/vbm/get_totals.m
