# Supplementary material for: Sex differences in olfactory cortex neuronal loss in aging
Source: Front Hum Neurosci. 2023 May 31;17:1130200. doi: 10.3389/fnhum.2023.1130200 (PMC10265738; doi:10.3389/fnhum.2023.1130200)
Supplement: Supplementary file 1 [file Table_1.docx]

***Supplementary Material***

**Supplementary Table 1**

Variables included in the study: comparison between males and females.

| **Variable** | **Males** | **Females** | ***p*** |
| --- | --- | --- | --- |
| Age (years) | 56.07 (19.57) | 54.91 (19.71) | 0.674 |
| Education (years) | 13.96 (4.62) | 13.01 (4.84) | 0.151 |
| TIV (l) | 1.46 (0.12) | 1.29 (0.09) | < 0.001 |
| OCV - Bilateral (ml) | 2.14 (0.28) | 1.97 (0.23) | < 0.001 |
| OCV - Left (ml) | 1.01 (0.14) | 0.93 (0.11) | < 0.001 |
| OCV - Right (ml) | 1.13 (0.15) | 1.04 (0.12) | < 0.001 |
